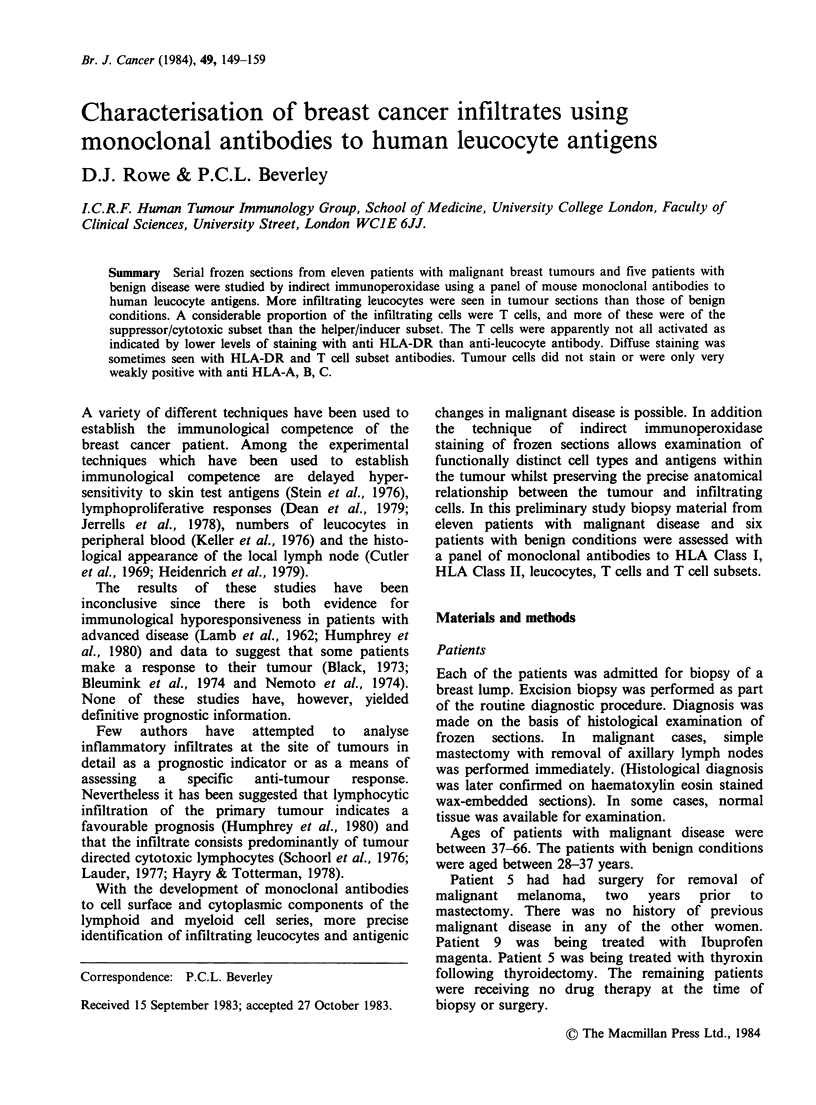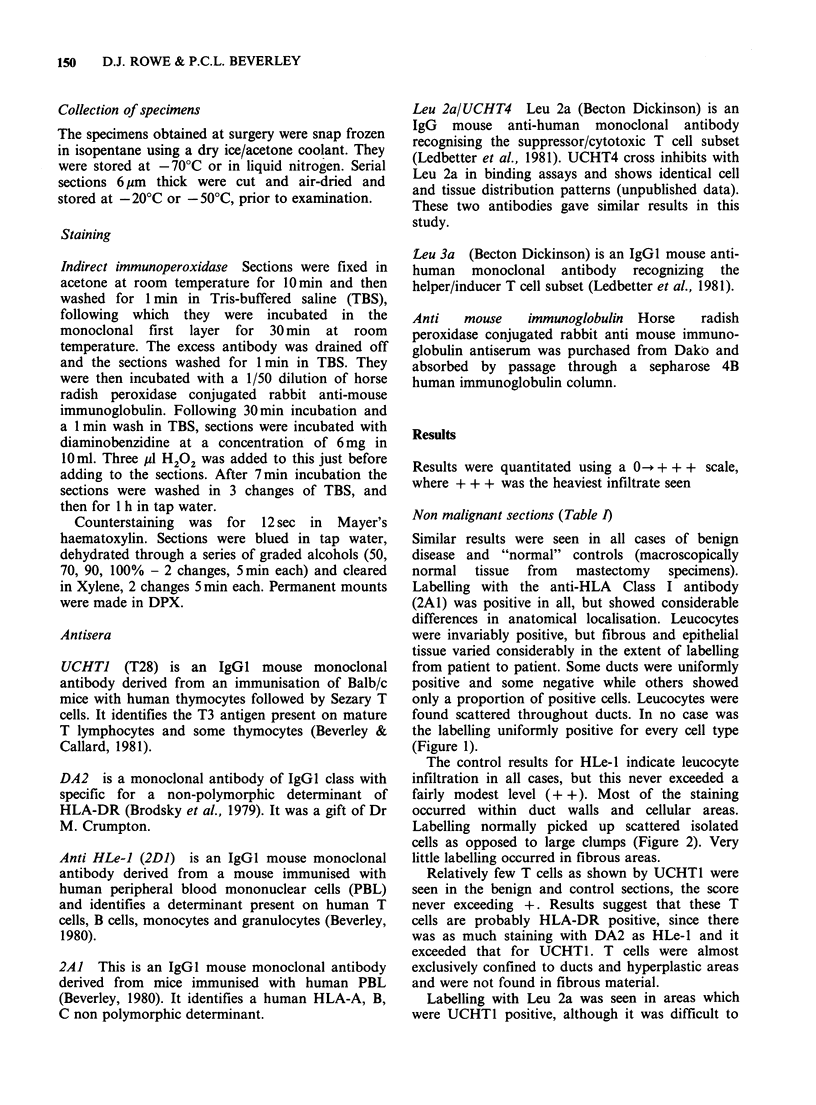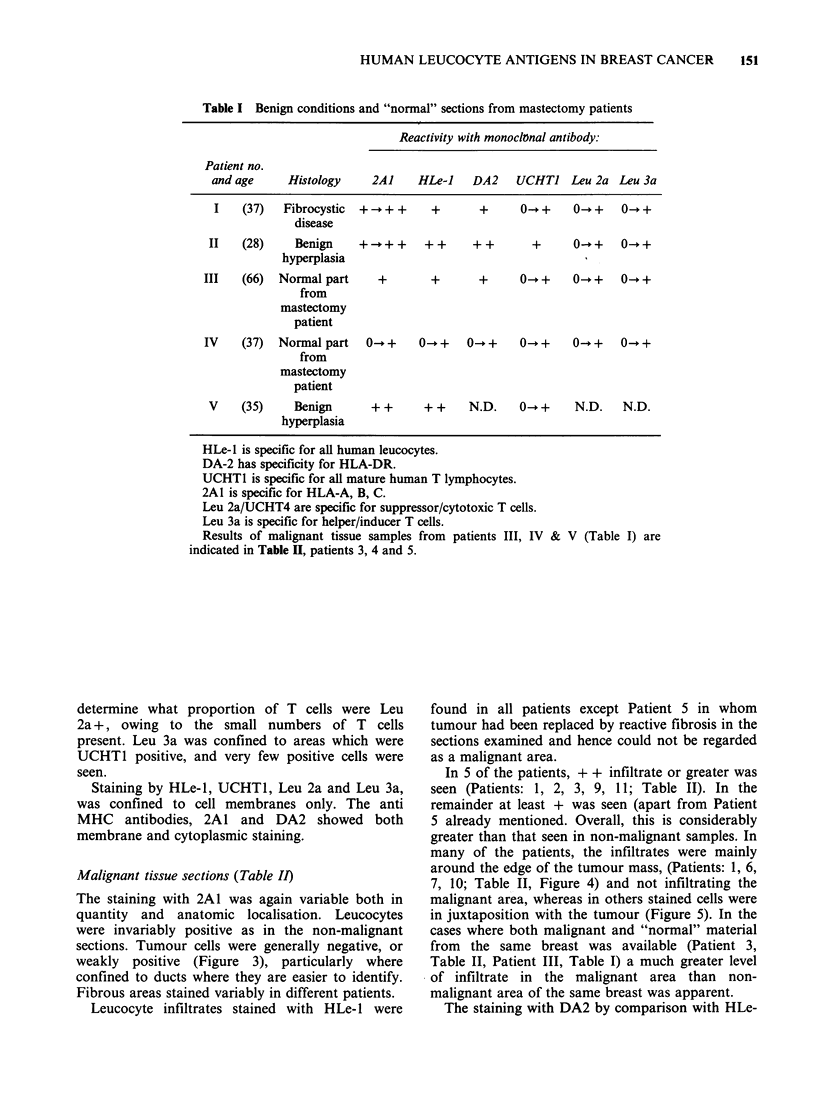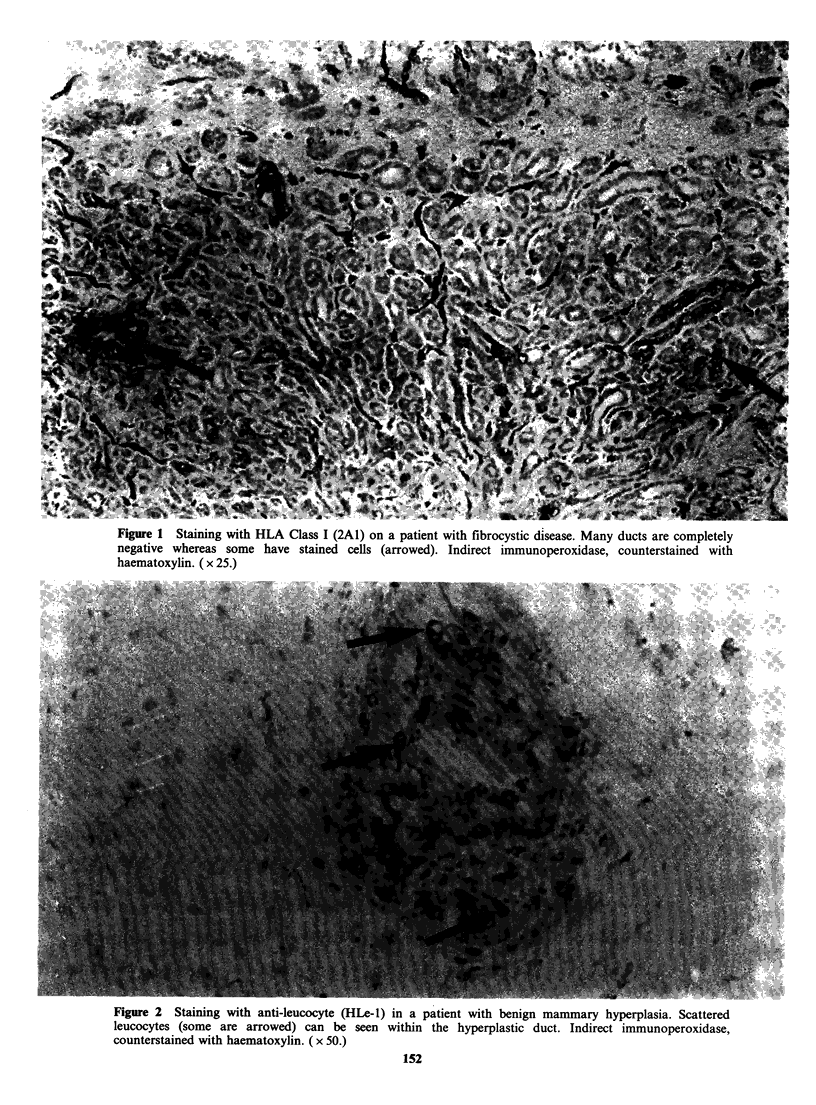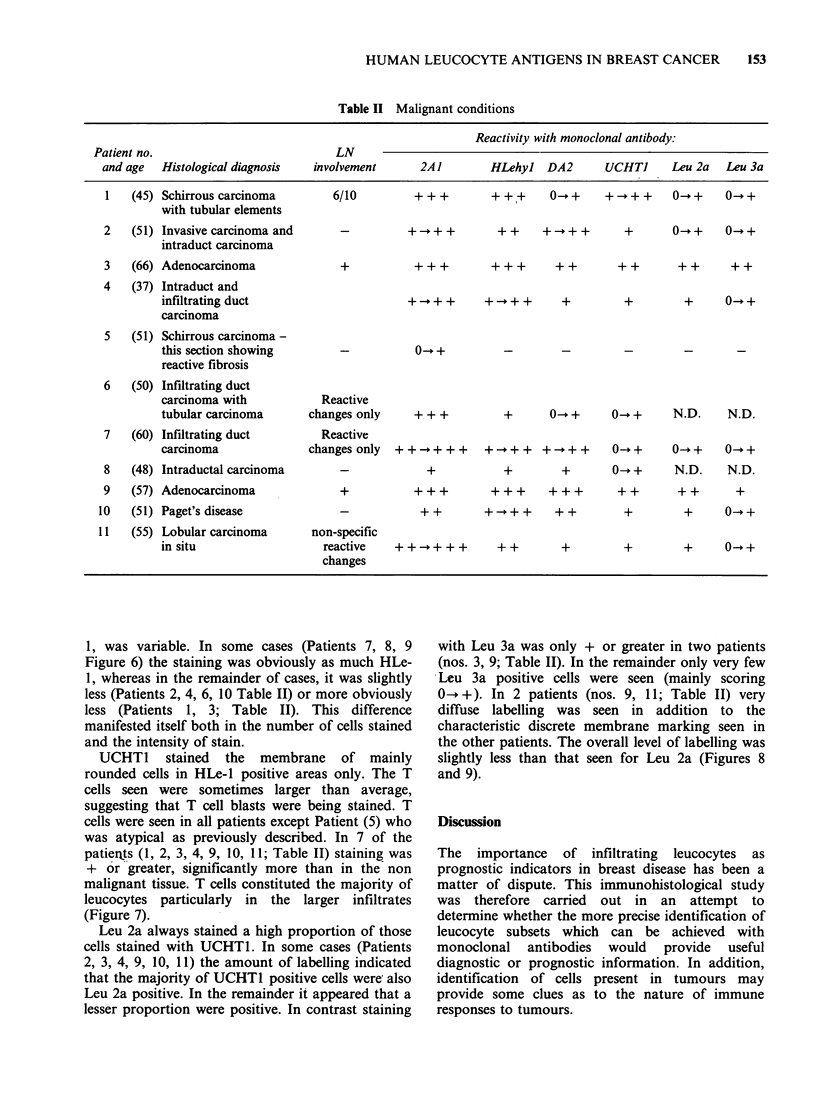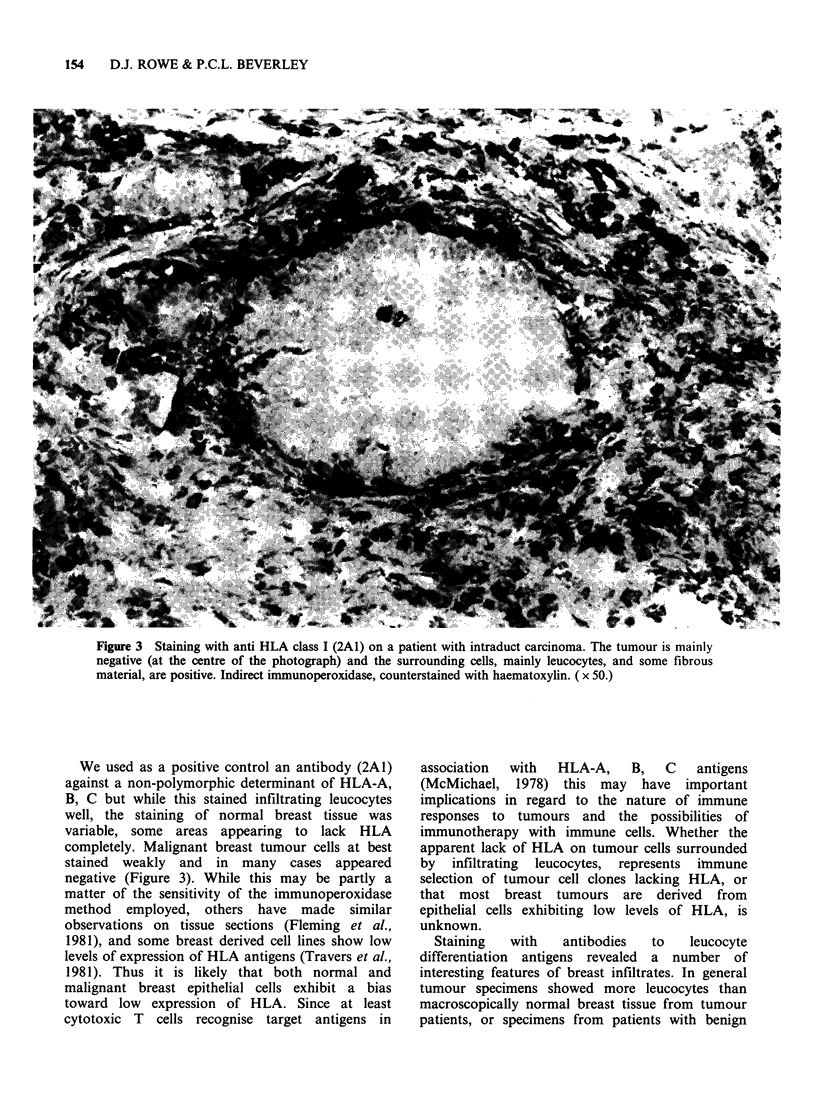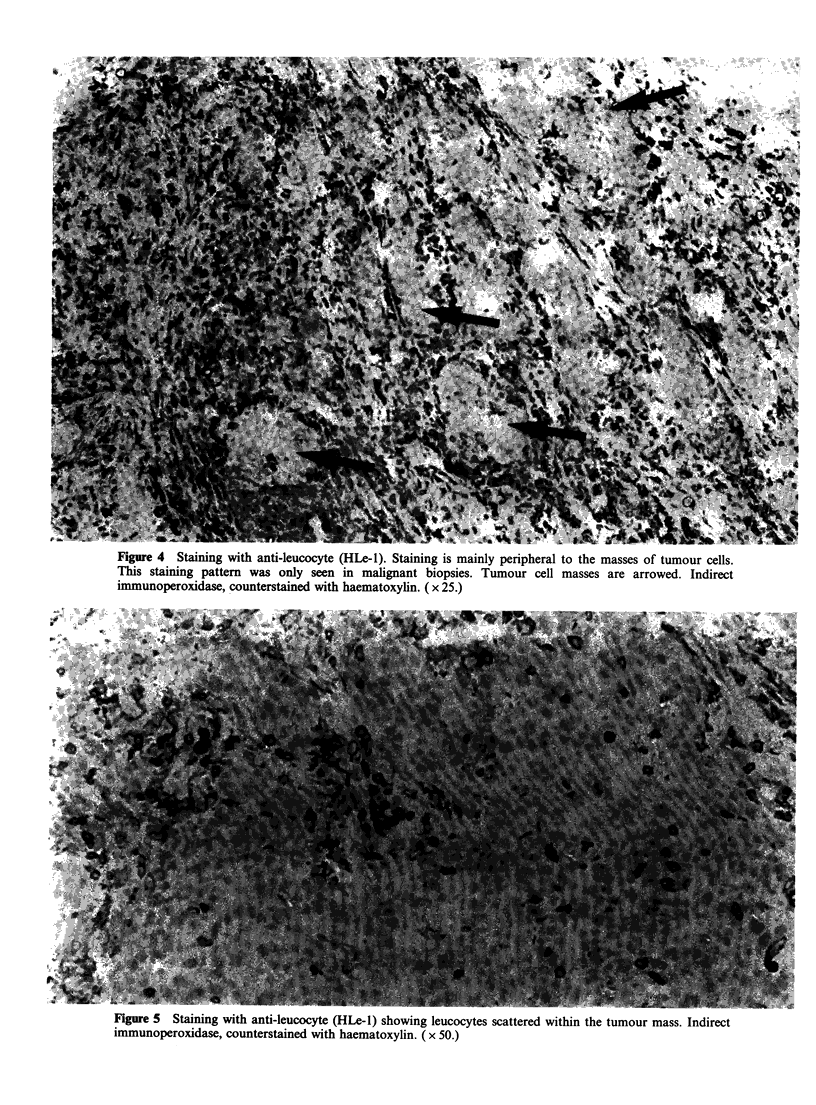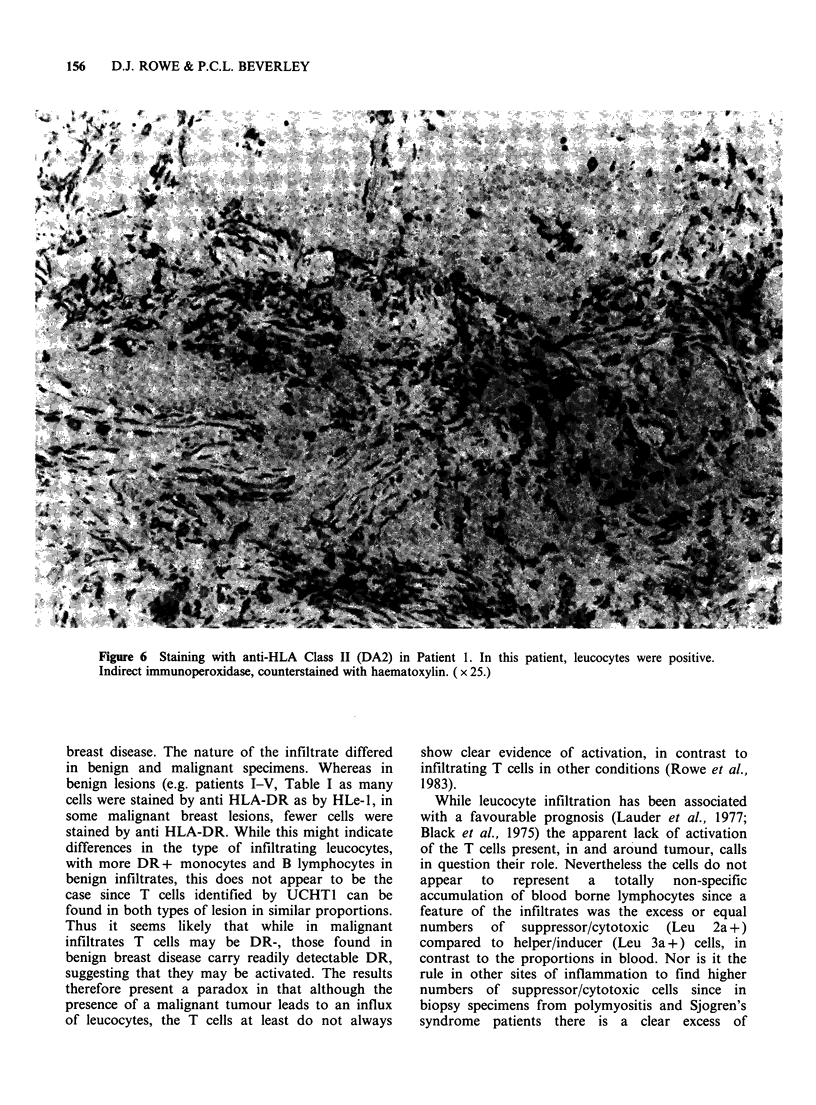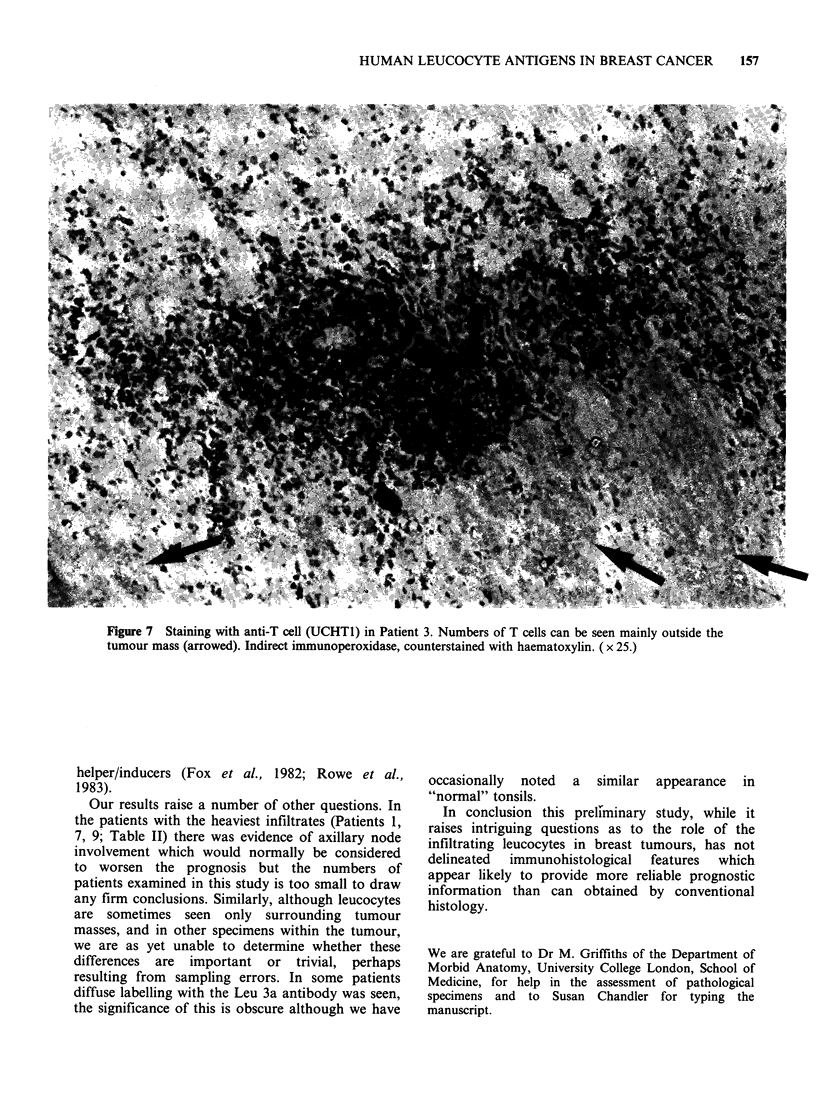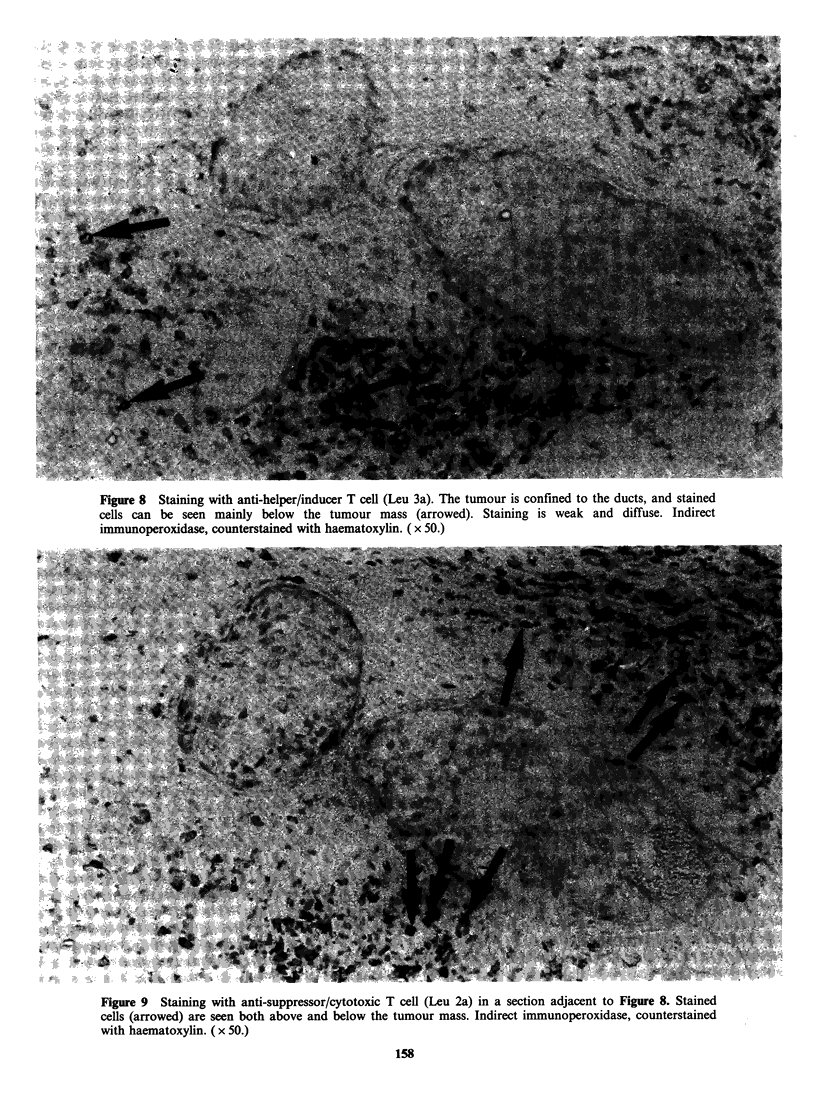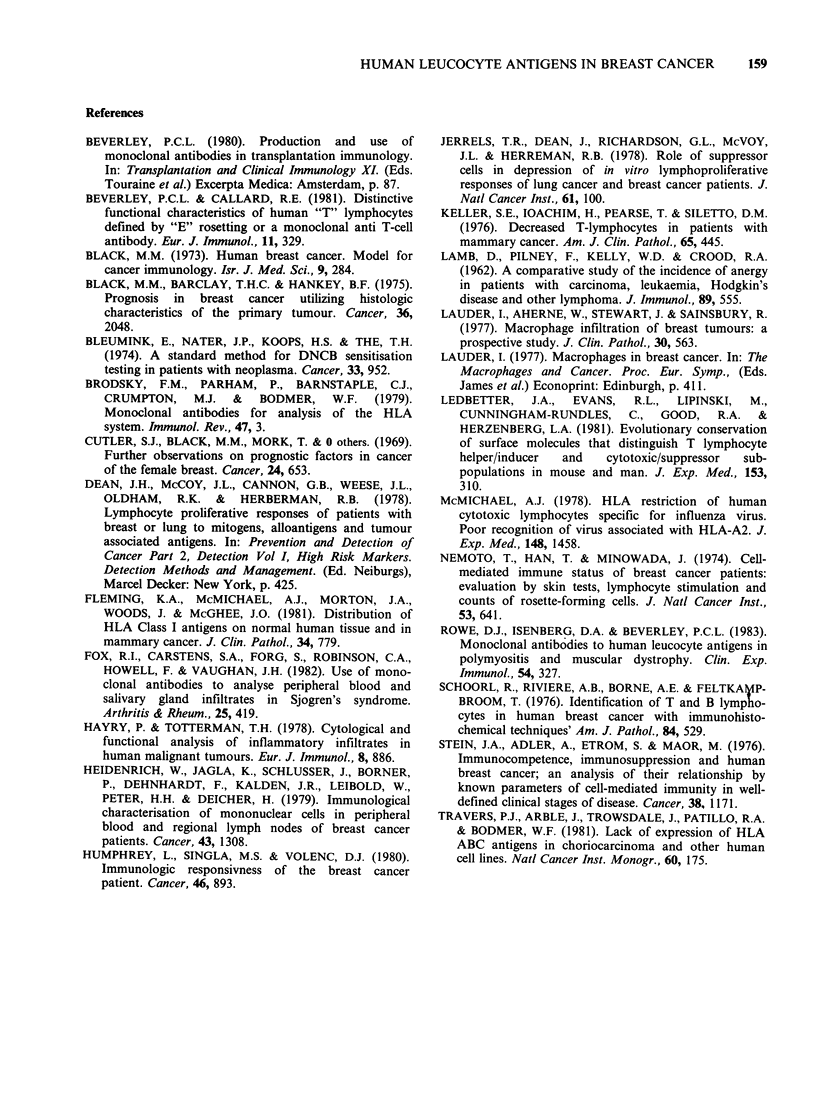# Characterisation of breast cancer infiltrates using monoclonal antibodies to human leucocyte antigens.

**DOI:** 10.1038/bjc.1984.27

**Published:** 1984-02

**Authors:** D. J. Rowe, P. C. Beverley

## Abstract

**Images:**


*Br. J. Cancer* (1984), **49**, 149–159

### Characterisation of breast cancer infiltrates using monoclonal antibodies to human leucocyte antigens

D.J. Rowe & P.C.L. Beverley

*I.C.R.F. Human Tumour Immunology Group, School of Medicine, University College London, Faculty of Clinical Sciences, University Street, London WC1E 6JJ.*

**Summary** Serial frozen sections from eleven patients with malignant breast tumours and five patients with benign disease were studied by indirect immunoperoxidase using a panel of mouse monoclonal antibodies to human leucocyte antigens. More infiltrating leucocytes were seen in tumour sections than those of benign conditions. A considerable proportion of the infiltrating cells were T cells, and more of these were of the suppressor/cytotoxic subset than the helper/inducer subset. The T cells were apparently not all activated as indicated by lower levels of staining with anti HLA-DR than anti-leucocyte antibody. Diffuse staining was sometimes seen with HLA-DR and T cell subset antibodies. Tumour cells did not stain or were only very weakly positive with anti HLA-A, B, C.

A variety of different techniques have been used to establish the immunological competence of the breast cancer patient. Among the experimental techniques which have been used to establish immunological competence are delayed hypersensitivity to skin test antigens (Stein *et al.*, 1976), lymphoproliferative responses (Dean *et al.*, 1979; Jerrells *et al.*, 1978), numbers of leucocytes in peripheral blood (Keller *et al.*, 1976) and the histological appearance of the local lymph node (Cutler *et al.*, 1969; Heidenrich *et al.*, 1979).

The results of these studies have been inconclusive since there is both evidence for immunological hyporesponsiveness in patients with advanced disease (Lamb *et al.*, 1962; Humphrey *et al.*, 1980) and data to suggest that some patients make a response to their tumour (Black, 1973; Bleumink *et al.*, 1974 and Nemoto *et al.*, 1974). None of these studies have, however, yielded definitive prognostic information.

Few authors have attempted to analyse inflammatory infiltrates at the site of tumours in detail as a prognostic indicator or as a means of assessing a specific anti-tumour response. Nevertheless it has been suggested that lymphocytic infiltration of the primary tumour indicates a favourable prognosis (Humphrey *et al.*, 1980) and that the infiltrate consists predominantly of tumour directed cytotoxic lymphocytes (Schoorl *et al.*, 1976; Lauder, 1977; Hayry & Totterman, 1978).

With the development of monoclonal antibodies to cell surface and cytoplasmic components of the lymphoid and myeloid cell series, more precise identification of infiltrating leucocytes and antigenic changes in malignant disease is possible. In addition the technique of indirect immunoperoxidase staining of frozen sections allows examination of functionally distinct cell types and antigens within the tumour whilst preserving the precise anatomical relationship between the tumour and infiltrating cells. In this preliminary study biopsy material from eleven patients with malignant disease and six patients with benign conditions were assessed with a panel of monoclonal antibodies to HLA Class I, HLA Class II, leucocytes, T cells and T cell subsets.

Correspondence: P.C.L. Beverley

Received 15 September 1983; accepted 27 October 1983.

### Materials and methods

### *Patients*

Each of the patients was admitted for biopsy of a breast lump. Excision biopsy was performed as part of the routine diagnostic procedure. Diagnosis was made on the basis of histological examination of frozen sections. In malignant cases, simple mastectomy with removal of axillary lymph nodes was performed immediately. (Histological diagnosis was later confirmed on haematoxylin eosin stained wax-embedded sections). In some cases, normal tissue was available for examination.

Ages of patients with malignant disease were between 37–66. The patients with benign conditions were aged between 28–37 years.

Patient 5 had had surgery for removal of malignant melanoma, two years prior to mastectomy. There was no history of previous malignant disease in any of the other women. Patient 9 was being treated with Ibuprofen magenta. Patient 5 was being treated with thyroxin following thyroidectomy. The remaining patients were receiving no drug therapy at the time of biopsy or surgery.

© The Macmillan Press Ltd., 1984

*Collection of specimens*

The specimens obtained at surgery were snap frozen in isopentane using a dry ice/acetone coolant. They were stored at −70°C or in liquid nitrogen. Serial sections 6 μm thick were cut and air-dried and stored at −20°C or −50°C, prior to examination.

*Staining*

*Indirect immunoperoxidase* Sections were fixed in acetone at room temperature for 10 min and then washed for 1 min in Tris-buffered saline (TBS), following which they were incubated in the monoclonal first layer for 30 min at room temperature. The excess antibody was drained off and the sections washed for 1 min in TBS. They were then incubated with a 1/50 dilution of horse radish peroxidase conjugated rabbit anti-mouse immunoglobulin. Following 30 min incubation and a 1 min wash in TBS, sections were incubated with diaminobenzidine at a concentration of 6 mg in 10 ml. Three μl $H_2O_2$ was added to this just before adding to the sections. After 7 min incubation the sections were washed in 3 changes of TBS, and then for 1 h in tap water.

Counterstaining was for 12 sec in Mayer's haematoxylin. Sections were blued in tap water, dehydrated through a series of graded alcohols (50, 70, 90, 100% – 2 changes, 5 min each) and cleared in Xylene, 2 changes 5 min each. Permanent mounts were made in DPX.

*Antisera*

*UCHT1* (T28) is an IgG1 mouse monoclonal antibody derived from an immunisation of Balb/c mice with human thymocytes followed by Sezary T cells. It identifies the T3 antigen present on mature T lymphocytes and some thymocytes (Beverley & Callard, 1981).

*DA2* is a monoclonal antibody of IgG1 class with specific for a non-polymorphic determinant of HLA-DR (Brodsky *et al.*, 1979). It was a gift of Dr M. Crumpton.

*Anti HLe-1* (*2D1*) is an IgG1 mouse monoclonal antibody derived from a mouse immunised with human peripheral blood mononuclear cells (PBL) and identifies a determinant present on human T cells, B cells, monocytes and granulocytes (Beverley, 1980).

*2A1* This is an IgG1 mouse monoclonal antibody derived from mice immunised with human PBL (Beverley, 1980). It identifies a human HLA-A, B, C non polymorphic determinant.

*Leu 2a/UCHT4* Leu 2a (Becton Dickinson) is an IgG mouse anti-human monoclonal antibody recognising the suppressor/cytotoxic T cell subset (Ledbetter *et al.*, 1981). UCHT4 cross inhibits with Leu 2a in binding assays and shows identical cell and tissue distribution patterns (unpublished data). These two antibodies gave similar results in this study.

*Leu 3a* (Becton Dickinson) is an IgG1 mouse anti-human monoclonal antibody recognizing the helper/inducer T cell subset (Ledbetter *et al.*, 1981).

*Anti mouse immunoglobulin* Horse radish peroxidase conjugated rabbit anti mouse immunoglobulin antiserum was purchased from Dako and absorbed by passage through a sepharose 4B human immunoglobulin column.

### Results

Results were quantitated using a 0→+++ scale, where +++ was the heaviest infiltrate seen

*Non malignant sections* (*Table I*)

Similar results were seen in all cases of benign disease and "normal" controls (macroscopically normal tissue from mastectomy specimens). Labelling with the anti-HLA Class I antibody (2A1) was positive in all, but showed considerable differences in anatomical localisation. Leucocytes were invariably positive, but fibrous and epithelial tissue varied considerably in the extent of labelling from patient to patient. Some ducts were uniformly positive and some negative while others showed only a proportion of positive cells. Leucocytes were found scattered throughout ducts. In no case was the labelling uniformly positive for every cell type (Figure 1).

The control results for HLe-1 indicate leucocyte infiltration in all cases, but this never exceeded a fairly modest level (++). Most of the staining occurred within duct walls and cellular areas. Labelling normally picked up scattered isolated cells as opposed to large clumps (Figure 2). Very little labelling occurred in fibrous areas.

Relatively few T cells as shown by UCHT1 were seen in the benign and control sections, the score never exceeding +. Results suggest that these T cells are probably HLA-DR positive, since there was as much staining with DA2 as HLe-1 and it exceeded that for UCHT1. T cells were almost exclusively confined to ducts and hyperplastic areas and were not found in fibrous material.

Labelling with Leu 2a was seen in areas which were UCHT1 positive, although it was difficult to

**Table I** Benign conditions and "normal" sections from mastectomy patients

| Patient no. and age | | Histology | *Reactivity with monoclonal antibody:* 2A1 | HLe-1 | DA2 | UCHT1 | Leu 2a | Leu 3a |
|---|---|---|---|---|---|---|---|---|
| I | (37) | Fibrocystic disease | +→++ | + | + | 0→+ | 0→+ | 0→+ |
| II | (28) | Benign hyperplasia | +→++ | ++ | ++ | + | 0→+ | 0→+ |
| III | (66) | Normal part from mastectomy patient | + | + | + | 0→+ | 0→+ | 0→+ |
| IV | (37) | Normal part from mastectomy patient | 0→+ | 0→+ | 0→+ | 0→+ | 0→+ | 0→+ |
| V | (35) | Benign hyperplasia | ++ | ++ | N.D. | 0→+ | N.D. | N.D. |

HLe-1 is specific for all human leucocytes.
DA-2 has specificity for HLA-DR.
UCHT1 is specific for all mature human T lymphocytes.
2A1 is specific for HLA-A, B, C.
Leu 2a/UCHT4 are specific for suppressor/cytotoxic T cells.
Leu 3a is specific for helper/inducer T cells.
Results of malignant tissue samples from patients III, IV & V (Table I) are indicated in **Table II**, patients 3, 4 and 5.

determine what proportion of T cells were Leu 2a+, owing to the small numbers of T cells present. Leu 3a was confined to areas which were UCHT1 positive, and very few positive cells were seen.

Staining by HLe-1, UCHT1, Leu 2a and Leu 3a, was confined to cell membranes only. The anti MHC antibodies, 2A1 and DA2 showed both membrane and cytoplasmic staining.

*Malignant tissue sections* (*Table II*)

The staining with 2A1 was again variable both in quantity and anatomic localisation. Leucocytes were invariably positive as in the non-malignant sections. Tumour cells were generally negative, or weakly positive (Figure 3), particularly where confined to ducts where they are easier to identify. Fibrous areas stained variably in different patients.

Leucocyte infiltrates stained with HLe-1 were found in all patients except Patient 5 in whom tumour had been replaced by reactive fibrosis in the sections examined and hence could not be regarded as a malignant area.

In 5 of the patients, ++ infiltrate or greater was seen (Patients: 1, 2, 3, 9, 11; Table II). In the remainder at least + was seen (apart from Patient 5 already mentioned. Overall, this is considerably greater than that seen in non-malignant samples. In many of the patients, the infiltrates were mainly around the edge of the tumour mass, (Patients: 1, 6, 7, 10; Table II, Figure 4) and not infiltrating the malignant area, whereas in others stained cells were in juxtaposition with the tumour (Figure 5). In the cases where both malignant and "normal" material from the same breast was available (Patient 3, Table II, Patient III, Table I) a much greater level of infiltrate in the malignant area than non-malignant area of the same breast was apparent.

The staining with DA2 by comparison with HLe-

**Figure 1** Staining with HLA Class I (2A1) on a patient with fibrocystic disease. Many ducts are completely negative whereas some have stained cells (arrowed). Indirect immunoperoxidase, counterstained with haematoxylin. (×25.)

**Figure 2** Staining with anti-leucocyte (HLe-1) in a patient with benign mammary hyperplasia. Scattered leucocytes (some are arrowed) can be seen within the hyperplastic duct. Indirect immunoperoxidase, counterstained with haematoxylin. (×50.)

**Table II** Malignant conditions

| Patient no. and age | | Histological diagnosis | LN involvement | Reactivity with monoclonal antibody: 2A1 | HLehy1 | DA2 | UCHT1 | Leu 2a | Leu 3a |
|---|---|---|---|---|---|---|---|---|---|
| 1 | (45) | Schirrous carcinoma with tubular elements | 6/10 | + + + | + + + | 0→+ | +→+ + | 0→+ | 0→+ |
| 2 | (51) | Invasive carcinoma and intraduct carcinoma | – | +→+ + | + + | +→+ + | + | 0→+ | 0→+ |
| 3 | (66) | Adenocarcinoma | + | + + + | + + + | + + | + + | + + | + + |
| 4 | (37) | Intraduct and infiltrating duct carcinoma | | +→+ + | +→+ + | + | + | + | 0→+ |
| 5 | (51) | Schirrous carcinoma – this section showing reactive fibrosis | – | 0→+ | – | – | – | – | – |
| 6 | (50) | Infiltrating duct carcinoma with tubular carcinoma | Reactive changes only | + + + | + | 0→+ | 0→+ | N.D. | N.D. |
| 7 | (60) | Infiltrating duct carcinoma | Reactive changes only | + +→+ + + | +→+ + | +→+ + | 0→+ | 0→+ | 0→+ |
| 8 | (48) | Intraductal carcinoma | – | + | + | + | 0→+ | N.D. | N.D. |
| 9 | (57) | Adenocarcinoma | + | + + + | + + + | + + + | + + | + + | + |
| 10 | (51) | Paget's disease | – | + + | +→+ + | + + | + | + | 0→+ |
| 11 | (55) | Lobular carcinoma in situ | non-specific reactive changes | + +→+ + + | + + | + | + | + | 0→+ |

1, was variable. In some cases (Patients 7, 8, 9 Figure 6) the staining was obviously as much HLe-1, whereas in the remainder of cases, it was slightly less (Patients 2, 4, 6, 10 Table II) or more obviously less (Patients 1, 3; Table II). This difference manifested itself both in the number of cells stained and the intensity of stain.

UCHT1 stained the membrane of mainly rounded cells in HLe-1 positive areas only. The T cells seen were sometimes larger than average, suggesting that T cell blasts were being stained. T cells were seen in all patients except Patient (5) who was atypical as previously described. In 7 of the patients (1, 2, 3, 4, 9, 10, 11; Table II) staining was + or greater, significantly more than in the non malignant tissue. T cells constituted the majority of leucocytes particularly in the larger infiltrates (Figure 7).

Leu 2a always stained a high proportion of those cells stained with UCHT1. In some cases (Patients 2, 3, 4, 9, 10, 11) the amount of labelling indicated that the majority of UCHT1 positive cells were also Leu 2a positive. In the remainder it appeared that a lesser proportion were positive. In contrast staining with Leu 3a was only + or greater in two patients (nos. 3, 9; Table II). In the remainder only very few Leu 3a positive cells were seen (mainly scoring 0→+). In 2 patients (nos. 9, 11; Table II) very diffuse labelling was seen in addition to the characteristic discrete membrane marking seen in the other patients. The overall level of labelling was slightly less than that seen for Leu 2a (Figures 8 and 9).

### Discussion

The importance of infiltrating leucocytes as prognostic indicators in breast disease has been a matter of dispute. This immunohistological study was therefore carried out in an attempt to determine whether the more precise identification of leucocyte subsets which can be achieved with monoclonal antibodies would provide useful diagnostic or prognostic information. In addition, identification of cells present in tumours may provide some clues as to the nature of immune responses to tumours.

**Figure 3** Staining with anti HLA class I (2A1) on a patient with intraduct carcinoma. The tumour is mainly negative (at the centre of the photograph) and the surrounding cells, mainly leucocytes, and some fibrous material, are positive. Indirect immunoperoxidase, counterstained with haematoxylin. ( × 50.)

We used as a positive control an antibody (2A1) against a non-polymorphic determinant of HLA-A, B, C but while this stained infiltrating leucocytes well, the staining of normal breast tissue was variable, some areas appearing to lack HLA completely. Malignant breast tumour cells at best stained weakly and in many cases appeared negative (Figure 3). While this may be partly a matter of the sensitivity of the immunoperoxidase method employed, others have made similar observations on tissue sections (Fleming *et al.*, 1981), and some breast derived cell lines show low levels of expression of HLA antigens (Travers *et al.*, 1981). Thus it is likely that both normal and malignant breast epithelial cells exhibit a bias toward low expression of HLA. Since at least cytotoxic T cells recognise target antigens in association with HLA-A, B, C antigens (McMichael, 1978) this may have important implications in regard to the nature of immune responses to tumours and the possibilities of immunotherapy with immune cells. Whether the apparent lack of HLA on tumour cells surrounded by infiltrating leucocytes, represents immune selection of tumour cell clones lacking HLA, or that most breast tumours are derived from epithelial cells exhibiting low levels of HLA, is unknown.

Staining with antibodies to leucocyte differentiation antigens revealed a number of interesting features of breast infiltrates. In general tumour specimens showed more leucocytes than macroscopically normal breast tissue from tumour patients, or specimens from patients with benign

**Figure 4** Staining with anti-leucocyte (HLe-1). Staining is mainly peripheral to the masses of tumour cells. This staining pattern was only seen in malignant biopsies. Tumour cell masses are arrowed. Indirect immunoperoxidase, counterstained with haematoxylin. (×25.)

**Figure 5** Staining with anti-leucocyte (HLe-1) showing leucocytes scattered within the tumour mass. Indirect immunoperoxidase, counterstained with haematoxylin. (×50.)

**Figure 6** Staining with anti-HLA Class II (DA2) in Patient 1. In this patient, leucocytes were positive. Indirect immunoperoxidase, counterstained with haematoxylin. ( × 25.)

breast disease. The nature of the infiltrate differed in benign and malignant specimens. Whereas in benign lesions (e.g. patients I–V, Table I as many cells were stained by anti HLA-DR as by HLe-1, in some malignant breast lesions, fewer cells were stained by anti HLA-DR. While this might indicate differences in the type of infiltrating leucocytes, with more DR+ monocytes and B lymphocytes in benign infiltrates, this does not appear to be the case since T cells identified by UCHT1 can be found in both types of lesion in similar proportions. Thus it seems likely that while in malignant infiltrates T cells may be DR-, those found in benign breast disease carry readily detectable DR, suggesting that they may be activated. The results therefore present a paradox in that although the presence of a malignant tumour leads to an influx of leucocytes, the T cells at least do not always show clear evidence of activation, in contrast to infiltrating T cells in other conditions (Rowe *et al.*, 1983).

While leucocyte infiltration has been associated with a favourable prognosis (Lauder *et al.*, 1977; Black *et al.*, 1975) the apparent lack of activation of the T cells present, in and around tumour, calls in question their role. Nevertheless the cells do not appear to represent a totally non-specific accumulation of blood borne lymphocytes since a feature of the infiltrates was the excess or equal numbers of suppressor/cytotoxic (Leu 2a+) compared to helper/inducer (Leu 3a+) cells, in contrast to the proportions in blood. Nor is it the rule in other sites of inflammation to find higher numbers of suppressor/cytotoxic cells since in biopsy specimens from polymyositis and Sjogren's syndrome patients there is a clear excess of

**Figure 7** Staining with anti-T cell (UCHT1) in Patient 3. Numbers of T cells can be seen mainly outside the tumour mass (arrowed). Indirect immunoperoxidase, counterstained with haematoxylin. (×25.)

helper/inducers (Fox *et al.*, 1982; Rowe *et al.*, 1983).

Our results raise a number of other questions. In the patients with the heaviest infiltrates (Patients 1, 7, 9; Table II) there was evidence of axillary node involvement which would normally be considered to worsen the prognosis but the numbers of patients examined in this study is too small to draw any firm conclusions. Similarly, although leucocytes are sometimes seen only surrounding tumour masses, and in other specimens within the tumour, we are as yet unable to determine whether these differences are important or trivial, perhaps resulting from sampling errors. In some patients diffuse labelling with the Leu 3a antibody was seen, the significance of this is obscure although we have occasionally noted a similar appearance in "normal" tonsils.

In conclusion this preliminary study, while it raises intriguing questions as to the role of the infiltrating leucocytes in breast tumours, has not delineated immunohistological features which appear likely to provide more reliable prognostic information than can obtained by conventional histology.

We are grateful to Dr M. Griffiths of the Department of Morbid Anatomy, University College London, School of Medicine, for help in the assessment of pathological specimens and to Susan Chandler for typing the manuscript.

**Figure 8** Staining with anti-helper/inducer T cell (Leu 3a). The tumour is confined to the ducts, and stained cells can be seen mainly below the tumour mass (arrowed). Staining is weak and diffuse. Indirect immunoperoxidase, counterstained with haematoxylin. ( × 50.)

**Figure 9** Staining with anti-suppressor/cytotoxic T cell (Leu 2a) in a section adjacent to **Figure 8.** Stained cells (arrowed) are seen both above and below the tumour mass. Indirect immunoperoxidase, counterstained with haematoxylin. ( × 50.)

**References**

BEVERLEY, P.C.L. (1980). Production and use of monoclonal antibodies in transplantation immunology. In: *Transplantation and Clinical Immunology XI.* (Eds. Touraine *et al.*) Excerpta Medica: Amsterdam, p. 87.

BEVERLEY, P.C.L. & CALLARD, R.E. (1981). Distinctive functional characteristics of human "T" lymphocytes defined by "E" rosetting or a monoclonal anti T-cell antibody. *Eur. J. Immunol.*, **11**, 329.

BLACK, M.M. (1973). Human breast cancer. Model for cancer immunology. *Isr. J. Med. Sci.*, **9**, 284.

BLACK, M.M., BARCLAY, T.H.C. & HANKEY, B.F. (1975). Prognosis in breast cancer utilizing histologic characteristics of the primary tumour. *Cancer*, **36**, 2048.

BLEUMINK, E., NATER, J.P., KOOPS, H.S. & THE, T.H. (1974). A standard method for DNCB sensitisation testing in patients with neoplasma. *Cancer*, **33**, 952.

BRODSKY, F.M., PARHAM, P., BARNSTAPLE, C.J., CRUMPTON, M.J. & BODMER, W.F. (1979). Monoclonal antibodies for analysis of the HLA system. *Immunol. Rev.*, **47**, 3.

CUTLER, S.J., BLACK, M.M., MORK, T. & 0 others. (1969). Further observations on prognostic factors in cancer of the female breast. *Cancer*, **24**, 653.

DEAN, J.H., McCOY, J.L., CANNON, G.B., WEESE, J.L., OLDHAM, R.K. & HERBERMAN, R.B. (1978). Lymphocyte proliferative responses of patients with breast or lung to mitogens, alloantigens and tumour associated antigens. In: *Prevention and Detection of Cancer Part 2, Detection Vol I, High Risk Markers. Detection Methods and Management.* (Ed. Neiburgs), Marcel Decker: New York, p. 425.

FLEMING, K.A., McMICHAEL, A.J., MORTON, J.A., WOODS, J. & McGHEE, J.O. (1981). Distribution of HLA Class I antigens on normal human tissue and in mammary cancer. *J. Clin. Pathol.*, **34**, 779.

FOX, R.I., CARSTENS, S.A., FORG, S., ROBINSON, C.A., HOWELL, F. & VAUGHAN, J.H. (1982). Use of monoclonal antibodies to analyse peripheral blood and salivary gland infiltrates in Sjogren's syndrome. *Arthritis & Rheum.*, **25**, 419.

HAYRY, P. & TOTTERMAN, T.H. (1978). Cytological and functional analysis of inflammatory infiltrates in human malignant tumours. *Eur. J. Immunol.*, **8**, 886.

HEIDENRICH, W., JAGLA, K., SCHLUSSER, J., BORNER, P., DEHNHARDT, F., KALDEN, J.R., LEIBOLD, W., PETER, H.H. & DEICHER, H. (1979). Immunological characterisation of mononuclear cells in peripheral blood and regional lymph nodes of breast cancer patients. *Cancer*, **43**, 1308.

HUMPHREY, L., SINGLA, M.S. & VOLENC, D.J. (1980). Immunologic responsivness of the breast cancer patient. *Cancer*, **46**, 893.

JERRELS, T.R., DEAN, J., RICHARDSON, G.L., McVOY, J.L. & HERREMAN, R.B. (1978). Role of suppressor cells in depression of *in vitro* lymphoproliferative responses of lung cancer and breast cancer patients. *J. Natl Cancer Inst.*, **61**, 100.

KELLER, S.E., IOACHIM, H., PEARSE, T. & SILETTO, D.M. (1976). Decreased T-lymphocytes in patients with mammary cancer. *Am. J. Clin. Pathol.*, **65**, 445.

LAMB, D., PILNEY, F., KELLY, W.D. & CROOD, R.A. (1962). A comparative study of the incidence of anergy in patients with carcinoma, leukaemia, Hodgkin's disease and other lymphoma. *J. Immunol.*, **89**, 555.

LAUDER, I., AHERNE, W., STEWART, J. & SAINSBURY, R. (1977). Macrophage infiltration of breast tumours: a prospective study. *J. Clin. Pathol.*, **30**, 563.

LAUDER, I. (1977). Macrophages in breast cancer. In: *The Macrophages and Cancer. Proc. Eur. Symp.*, (Eds. James *et al.*) Econoprint: Edinburgh, p. 411.

LEDBETTER, J.A., EVANS, R.L., LIPINSKI, M., CUNNINGHAM-RUNDLES, C., GOOD, R.A. & HERZENBERG, L.A. (1981). Evolutionary conservation of surface molecules that distinguish T lymphocyte helper/inducer and cytotoxic/suppressor subpopulations in mouse and man. *J. Exp. Med.*, **153**, 310.

McMICHAEL, A.J. (1978). HLA restriction of human cytotoxic lymphocytes specific for influenza virus. Poor recognition of virus associated with HLA-A2. *J. Exp. Med.*, **148**, 1458.

NEMOTO, T., HAN, T. & MINOWADA, J. (1974). Cell-mediated immune status of breast cancer patients: evaluation by skin tests, lymphocyte stimulation and counts of rosette-forming cells. *J. Natl Cancer Inst.*, **53**, 641.

ROWE, D.J., ISENBERG, D.A. & BEVERLEY, P.C.L. (1983). Monoclonal antibodies to human leucocyte antigens in polymyositis and muscular dystrophy. *Clin. Exp. Immunol.*, **54**, 327.

SCHOORL, R., RIVIERE, A.B., BORNE, A.E. & FELTKAMP-BROOM, T. (1976). Identification of T and B lymphocytes in human breast cancer with immunohistochemical techniques' *Am. J. Pathol.*, **84**, 529.

STEIN, J.A., ADLER, A., ETROM, S. & MAOR, M. (1976). Immunocompetence, immunosuppression and human breast cancer; an analysis of their relationship by known parameters of cell-mediated immunity in well-defined clinical stages of disease. *Cancer*, **38**, 1171.

TRAVERS, P.J., ARBLE, J., TROWSDALE, J., PATILLO, R.A. & BODMER, W.F. (1981). Lack of expression of HLA ABC antigens in choriocarcinoma and other human cell lines. *Natl Cancer Inst. Monogr.*, **60**, 175.